# Research on Performance Optimization and Application in Smart Home for Hyperledger Fabric

**DOI:** 10.3390/s22093222

**Published:** 2022-04-22

**Authors:** Lanfang Ren, Huachun Zhou, Xiaoyong Hang, Bo Yang, Li Su

**Affiliations:** 1 School of Electronic and Information Engineering, Beijing Jiaotong University, Beijing 100044, China; 2 China Mobile Communication Corporation Research Institute, Beijing 100053, China; renlanfang@chinamobile.com (L.R.); hangxiaoyong@chinamobile.com (X.H.); yangbo@chinamobile.com (B.Y.); suli@chinamobile.com (L.S.)

**Keywords:** security of smart homes, access control for smart homes, security control for smart homes, blockchain, fabric

## Abstract

With the popularity of smart home services, smart home devices are also increasing significantly. At the same time, security problems of smart home services are becoming more and more important. With the characteristics of non-tampering and multi-party consensus mechanism, Blockchain technology provides powerful capabilities in security protection. In this paper, we introduce the widely used permission Blockchain Fabric for smart home services. As the high requirements of performance, we firstly study the methods of performance optimization for Fabric. Then, a Fabric-based smart home security control system is designed. Based on this system, the smart home system is able to provide access control and security control for smart home devices. The experimental results show that the system works best when the number of concurrent registrations of smart home device is under 6000.

## 1. Introduction

Smart homes have become popular in recent years and smart home devices are also increasing significantly. At this moment, the security problems of smart home services are becoming more and more important. Considering the security characteristics of non-tampering and multi-party consensus mechanism, Blockchain technology is useful for smart home services.

In the Blockchain system, all participants maintain a ledger file based on a consensus mechanism [1,2]. The permission chain of Hyperledger Fabric is currently the most popularly and widely used in different industries [3,4,5,6]. In the Fabric system, the nodes can be grouped into three types: endorsement node (endorser), ordering node (orderer) and committing node (committer). With these three types of nodes, the entire transaction procedure of Fabric includes three stages: endorsement, ordering and committing.

However, due to the limited throughput and high latency, Fabric suffers from performance problems when directly applied to smart home scenarios [7,8,9,10]. There are many works about performance optimization, but most of them focus on the optimization of consensus algorithms, on-chain and off-chain expansion, side-chain mechanisms, cross-chain mechanisms, and new types of consensus [11,12].

In this paper, we firstly analyze the key performance factors of each stage and summarize the existing optimization methods. Then, we propose some new optimizations from different stages of Fabric. Combining with the security risks of smart home services, we design a Fabric-based security control system. The experiment results show that the optimized Fabric is useful for smart home scenarios. The various sections of the paper are structured as per guideline [13], given in the paper.

## 2. Related Works

A Blockchain-based smart home access control scheme is proposed to provide sensitive privacy data and shared data protection for smart home scenarios [14].

A Blockchain-based solution for secure smart home systems is a combined Hyperledger fabric and Hyperledger composer. The system architecture contains four layers: Cloud storage, Hyperledger fabric, Hyperledger composer, and a smart home layer. By mapping the attributes of a smart home to those from the Hyperledger composer, it allows for a customized, designed-for-purpose solution, which can meet the security requirements for IoT-based smart homes [15].

Further, Blockchain has been considered with the concept of Smart Contracts. The scheme implements access control strategies through smart contracts of Fabric. With the strategies of user attribute, a hybrid access control model is designed [16]. Combining the power of Blockchain and public-key cryptography ensures secure authentication and identification of people and devices [17].

This paper not only considers the application of Blockchain in smart home scenarios, but also considers the high-performance requirements of Blockchain in smart home scenarios firstly.

## 3. Discussion of Performance in Fabric

**Figure 1:**
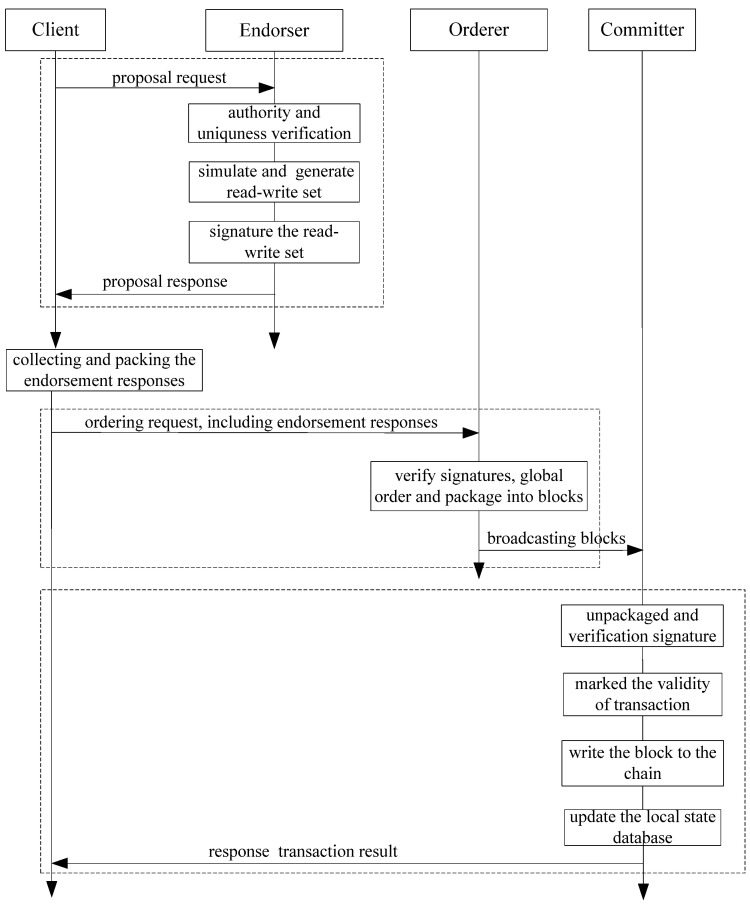
The flow of specific Fabric transaction processing.

The specific transaction processing of Fabric is shown in Figure 1.

1. Endorsement stage

The client sends a transaction request to all endorsers of the system. After receiving the request, the endorser verifies the identity and the authority of the client, and confirms the uniqueness based on the transaction ID. After that, the read-write set is generated based on the simulation execution of the local state database. Finally, the endorsers return the signature proposal responses to the client.

After receiving the endorsement response from endorsers, the client needs to verify whether the responses from different endorsers are exactly the same as each other. If not, this request is invalid and the client needs to re-initiate it. After that, the client packages the responses and sends as an ordering request to the orderer.

2. Ordering stage

When receiving the ordering request, the orderer firstly verifies the signature of the client and endorsers. Then, the orderer globally orders the transaction request based on the preset ordering mechanism. Finally, the orderer packages the transaction into certain sizes of blocks and broadcasts them to different committers.

3. Committing stage

In this stage, the committer actively receives the block broadcasted by the orderer and verifies the format and signature of the block. Then, the committer unpacks the block and examines the signatures of the endorsers. The committer also needs to verify the read-write set by accessing the local state database and mark the transaction as valid or invalid, according to the verification result of the read-write set. After that, the blocks are written to the chain while updating the local state database, according the write set of the valid transaction.

### 3.1. Key Factors

1. Endorsement Stage

According to the processing of endorsement stage in Fabric, the key factors of performance include:

- Complexity of algorithm: As the signature follows the entire Fabric process, the algorithm complexity of the signature directly influences the efficiency of the endorsement.
- Complexity of endorsement: The endorsement strategy specifies how many endorsers participated in the endorsement process. The more endorsers engage in the process, the longer the processing delay.
- Performance of database: In this stage, the endorser needs to query the state database and simulate the execution of transactions. The performance of the state database directly impacts endorsement efficiency.
- The consistency of ledger: The consistency of the ledger in different endorsers determines the validation of the endorsement responses. If they are inconsistent with each other, the re-endorsement is needed.
- The processing mode of transaction: The processing mode of endorsement responses affect the efficiency of the endorsement stage. Whether the endorsement request is processed in parallel or serially results in different influences on performance.
- The system performance: The peers with high CPU and big memory have faster processing efficiency.

2. Ordering Stage

In the ordering stage, the key factors impacting performance are as follows:

- Complexity of algorithm: Similar to the endorsement process, after receiving the ordering request, the orderer needs to verify the validity of the signature. Therefore, the complexity of the signature algorithm directly influences the performance.
- Size of message: After receiving the client’s request, the orderer needs to order all of the proposals globally. Actually, the orderer receives ordering requests from multiple clients at the same time. Therefore, the size of the proposal message will impact the ordering efficiency. Obviously, the smaller the message, the quicker the ordering.
- Ordering mechanism: Different ordering mechanisms have different effects on the ordering results and efficiency.
- The rate of block generation: The rate of block generation influences the performance of the system. If the time interval between block generations is too short, it may cause system congestion. And if the time interval is too long, it will cause waste of idle system resources. Intervals that are too long or too short can affect system performance.
- Block broadcast strategy: When the orderer broadcasts the packaged blocks to the committers, different broadcast strategies (for example, real-time broadcast, set threshold batch broadcast) also have an impact on the processing.
- Range of block broadcast: The orderer broadcasts a block to the committers in the system. The larger the broadcast range, the longer the delay.
- Protocol of block broadcast: The protocol of block broadcasting also impacts the efficiency.

3. Committing Stage

In the committing stage, the key factors impacting performance are as follows:

- Complexity of algorithm: After receiving the blocks, the committer firstly verifies the signature. Therefore, the complexity of the signature algorithm directly influences the efficiency.
- The size of block: The size of the block itself and the number of transactions contained in it determine the workload of the committer. The larger the blocks, the longer the processing delay.
- Performance of database: When verifying the transaction, the committer needs to access the local ledger database. The performance of the database seriously influences the processing efficiency.
- The mechanism of verification: In the committing stage, the mechanism of transaction verification greatly determines the workload and efficiency of the committers. Specifically, when the committer needs to verify all of the transactions in blocks, parallel or serial verification processing have different influences on the Fabric performance.

4. Discussion

In each stage of Fabric, different factors have an influence on performance. When performing performance optimization, multiple factors should be considered together. Furthermore, the factor with high weights is suggested firstly. According to theoretical analysis and experimental results, the factors of performance can be classified into three weights: high, middle and low (detailed information shown in Table 1).

### 3.2. Existing Solutions

1. Endorsement Stage

In endorsement stage, the endorser just simulates execution of the transaction. Compared with others, the endorsement stage has a smaller impact on performance. From the literature, reference [18] proposes to distinguish the transaction request according to the read or write operation and simplifies the processing of the read operation, and then improves the efficiency of endorsement.

Further, according to the key factors of performance in this stage, methods of simplifying algorithm complexity, optimization endorsement strategy and modifying the ledger mechanism are important for enhancing the efficiency of this stage.

2. Ordering Stage

In the ordering stage, the result determines the sequence of transaction verification and the workload of the committers. From the literature, reference [19] proposes two ordering optimization methods. One minimizes the message size by extracting transaction ID and ordering, based on the transaction ID. Another way is adjusting the processing mechanism from serial to parallel. From the literature, reference [20] describes a transaction rearrangement mechanism. By establishing a transaction-directed conflict graph, conflicting invalid transactions are identified as early as possible. This solution reduces the number of ordering transactions, and especially reduces the workload of committers in the subsequent process.

3. Committing Stage

The committing stage takes the longest time and has the greatest impact on performance. The existing solutions in this stage mainly focus on improving the database performance and optimizing the mechanism of transaction verification. In Fabric, the local state database supports both LevelDB and CounchDB, but the experimental result in reference [21] finds that the efficiency of CounchDB is relatively slower than LevelDB. Therefore, LevelDB is recommended and preferred over CounchDB in Fabric.

As for optimizing the mechanism of transaction verification, reference [22] proposes using a memory hash table to replace the local state database and also uses processing in parallel. This can enhance the efficiency of data query and improve the performance of transaction committing. Reference [21] minimizes the data size of the gossip protocol by broadcasting the calculated hash information rather than the entire new block. Moreover, committers receive the new block from other nodes, not only from the orderer. This scheme reduces the transmission of the entire block in the network. Reference [23] introduces a parsing node to classify transactions according to different smart contracts. Then, the parsing node sends different transactions to different committers to verify. This proposal greatly reduces the workload of the committer and improves the overall transaction processing performance.

### 3.3. New Proposals

1. Endorsement Stage

**Figure 2:**
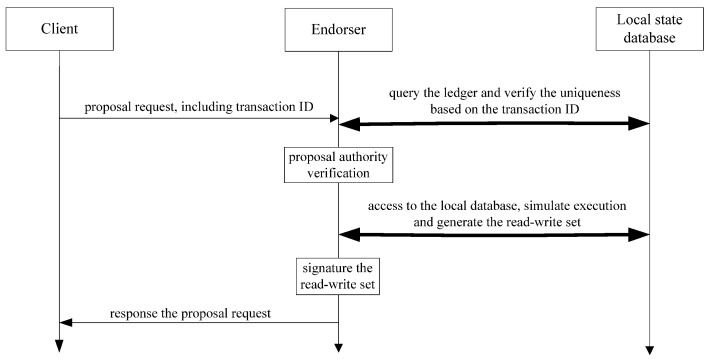
Original endorsement processing flow of Fabric.

The original Fabric endorsement processing is shown Figure 2. After receiving the proposal request, the endorser firstly queries the local state database and confirms the uniqueness based on the transaction ID. Then, the endorser verifies the authority of the client. This mechanism may cause a DoS attack on the endorser by malicious clients continuously sending a large number of proposals and exhausting the resources of the endorser [24,25].

In the endorsement stage, the endorser needs to access the local ledger database at least twice for each transaction request. Once for verifying the uniqueness and the other for simulating the execution and then generating the read-write set. This process can be considered for optimization.

Optimizations:

(a) Verify authority firstly, then uniqueness.

When receiving the transaction proposal request, the endorser verifies the authority of the client firstly. Then, the endorser performs uniqueness verification and simulates execution only for those authorized clients. This can reduce the possibility of being attacked by malicious users and, therefore, improve the security of Fabric.

(b) Merging two queries from the local database into one.

After completing the uniqueness verification, the endorser simulates execution and generates the read-write set by querying the state database just once. Furthermore, a temporary caching and paralleling operation can be considered in this stage. For example, the endorser initiates the endorsement based on some conditions, such as the number of requests, the size of the message, and the delay of previous processing, etc. The performance of Fabric can be improved by reducing the interaction with the local state database.

**Figure 3:**
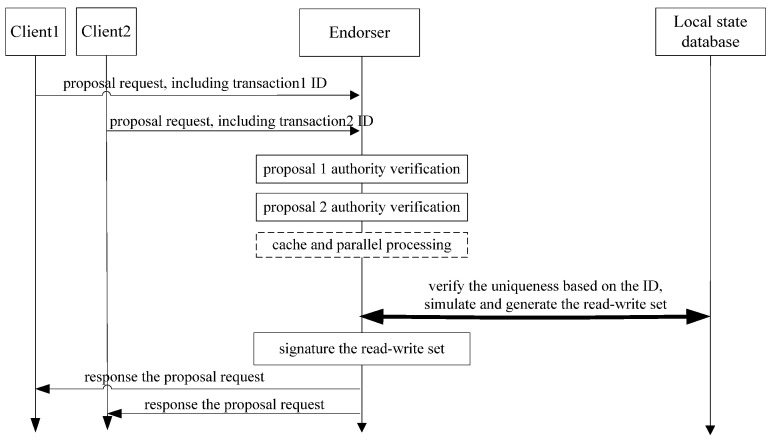
Optimized endorsement processing flow of Fabric.

The optimized endorsement process is shown in Figure 3.

2. Ordering Stage

In the ordering stage, the result of ordering determines the sequence of subsequent transaction verifications. In fact, different ordering results lead to different validation results in the committing stage. For example, the following are five pieces of ordered transactions in a block, T1–T5.

T1: W (k1, v1′), W (k2, v2′)

T2: R (k1), W (k3, v3′)

T3: W (k2, v2′’)

T4: W (k2, v2‴), R (k2) 

T5: W (k6, v6′), R (k5)

Here, W represents the write set, R represents the read set, k represents the key value of the transaction, and v represents the current true value of k.

In the committing stage, the committer confirms the transaction based on the read-write set. Firstly, the committer checks whether the read set is valid in this transaction. When the read set is valid, the committer updates the value of the key based on that write set of the transaction. If there is no read set but only a write set in a transaction, then the transaction is defaulted valid.

The verification processes of the above transactions are:

- T1: the top one without read set and the transaction of T1 is marked as valid.
- T2: the transaction is invalid, because T1 has changed k1.
- T3: without read set, marked valid directly.
- T4: the transaction is invalid, because it reads k2 but the T1 has changed k2.
- T5: the transaction is valid, because it reads k5 and k5 without being modified by other transactions.

Therefore, the committing results of the ordered transaction T1–T5 are: T1: valid; T2: invalid; T3: valid; T4: invalid; T5: valid.

With this ordering mechanism, a large number of invalid transactions exist in the block and are a waste of Blockchain resources.

The orderer only orders all the received transactions in the stage of ordering, and does not need to do any other processing of the transaction. This result in the conflicted transactions cannot be identified in a timely manner. After that, all the ordered transactions will be confirmed in the committing stage. A lot of verification work for the committer extends the whole processing delay of committing. In addition, more and more invalid transactions will be written on the Blockchain.

Optimizations:

(a) Optimize the ordering mechanism based on the read-write set of the transaction.

The transaction ordering for the orderer is not based on the receiving time any more, but based on the read-write set of the transaction. The specific proposal is as follows:

- First read then write: the transaction with the read set is ordered before the write set;
- Write last: the transaction with only the write set is ordered last.

According to this method, the new ordering result of T1–T5 is:

T2: R (k1), W (k3, v3′)

T4: W (k2, v2‴), R (K2)

T5: W (k6, v6′), R (k5)

T1: W (k1, v1′), W (k2, v2′)

T3: W (k2, v2′)

Then, the verification result of the transactions is: T1: effective; T2: effective; T3: effective; T4: effective; T5: effective.

This proposal can obviously improve the validation of the transaction and enhance the effectiveness of Blockchain.

(b) First confliction detecting then ordering.

Before ordering, the orderer needs to perform transaction confliction detecting by analyzing the read-write sets of transactions. The orderer marks the conflicted transactions with invalid tags, rejects the order and discards them. The orderer sends a conflicting response to the clients of the discarded transactions. Also, for the conflicting transaction, the orderer firstly marks invalid tag, then normally processing by ordering and packaging in to blocks.. Later in the committing stage, the committer will ignore the invalid transactions. This method can reduce the workload of the committer and enhance the performance of Fabric.

**Figure 4:**
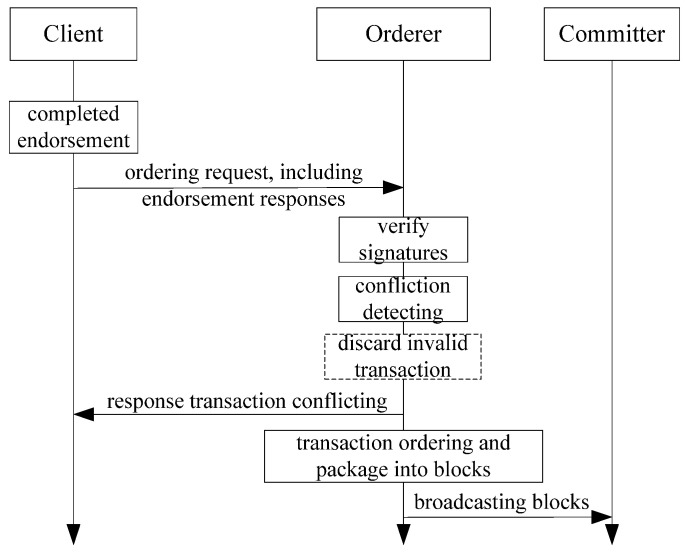
Optimized ordering processing flow of Fabric.

The optimized ordering process is shown in Figure 4.

3. Committing Stage

The existing committing process has the following improvement points:

- In Fabric, the committers need to verify all transactions in a new block. For example, if there are N committers and 10 transactions in each block, these need to be verified 10×N times for each block. This method is a waste of resources and influences the performance of Fabric.
- After finishing the verification, each of the committers needs to write the local ledger and update the local state database. Repeated storage consumes a lot of resources and the synchronization of different ledger files is also a problem.

Optimization:

(a) Transaction grouping verification

Based on the grouping rules, the orderer distinguishes the transactions and packages different groups into different blocks. After that, the orderer sends the different blocks to some of the committers in the system. In this case, committers do not need to verify all blocks in the system. The performance of Fabric can be enhanced.

(b) Splitting the ledger

Based on grouped transaction, the committers only need to confirm a part of the transaction, and also only need to write a part of the ledger file.

(c) Merging one Ledger

In this proposal, only one node is selected to write and keep the ledger file, which can be the order or another authority committer. In this case, when completing the transaction verification in the confirming stage, committers just need to send back the results to this special node. The ledger file is updated by that special node, according to the committing results.

4. Evaluation

Performance optimizations of Fabric in different stages are described above. In the endorsement stage, the solution modifies the transaction processing flow and the endorser needs to temporarily cache and process the proposal requests in parallel. This proposal has no effect on the orderer and committer. In the ordering stage, by confliction detecting and optimizing the order mechanism, the proposal improves the validation of transactions on the chain and reduces the workload of the committer. In the processing of committing, the orderer packages transactions in groups and sends the different blocks to different committers. The committer only confirms a part of the transaction, and just stores a part of the ledger file. Furthermore, there can be only one special node being selected to write and store the ledger file; this proposal improves system processing performance while utilizing resources efficiently.

All of the above optimization proposals can be used individually or in combination with others to improve the performance of Fabric.

## 4. Design of Smart Home Security Control System Based on Fabric

Smart homes entail a comprehensive communication scenario of computer, network and Internet of Things, artificial intelligence and big data technologies. With the development of smart home services, homeowners can remotely control and interact with home devices through smart home terminals and controllers intelligently. More and more smart home applications create an efficient, comfortable and secure personalized home life.

With the popularity of smart home services, types and numbers of smart home devices are also increasing significantly. At the same time, security problems and security risks about smart home services are becoming more and more important. It is well known that different smart home devices have different operating systems and applications and there are various security risks involved with smart home devices. Actually, the security of smart home devices determines the whole system security of the smart home service. For example, modifying the configuration information of smart home devices maliciously could result in serious security incidents.

### 4.1. System Architecture

**Figure 5:**
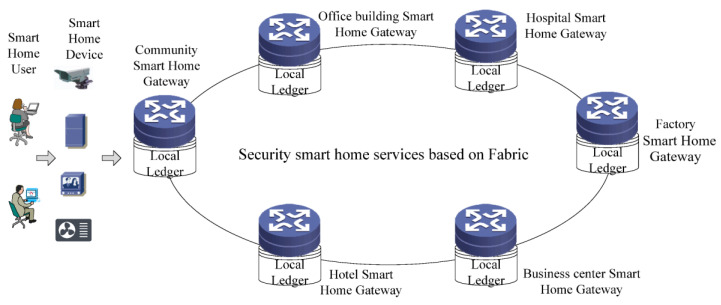
The network model of smart home security control services based on Fabric.

With the characteristics of non-tampering and multi-party consensus mechanism, Blockchain technology provides powerful capabilities in security protection [26,27,28,29]. This paper proposes a smart home security control service based on Fabric. The network model of this system is shown in Figure 5.

**Figure 6:**
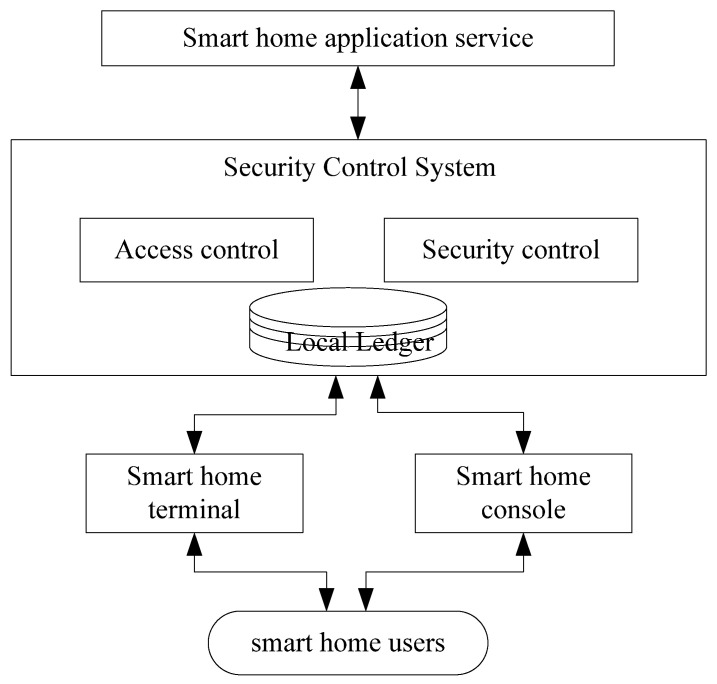
The architecture of security control system.

In this model, all smart home gateways in the network construct a security control system based on Fabric. Each gateway is a peer of Fabric and has its own local ledger and the smart home device is the client of Fabric. In the system, the smart home gateway provides access controls and security control for smart home devices, according to the certificate and key device information of the device. The information is stored on the chain after the consensus of other gateways. The architecture of the security control system is shown in Figure 6.

In the system, the access control module provides certificate-based accessing control for devices. The security control module is responsible for the integrity verification of the key information during the running processing of smart home devices. It also disconnects the tampered device from the smart home services. The device’s certificates and key information are securely stored in Fabric.

### 4.2. Access Control

With the purpose of accessing the smart home system successfully, the device manufacturer needs to apply a certificate for each of the smart home devices from the CA in advance. Access control processing includes two aspects: registering and accessing control.

**Figure 7:**
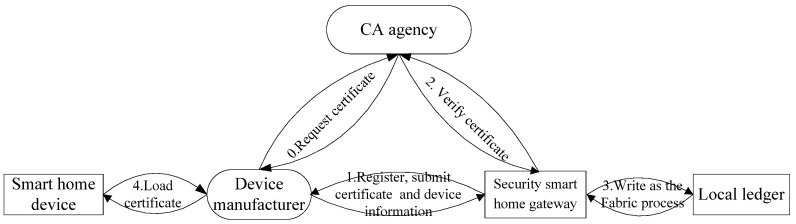
Registering of smart home device.

1. Registering:

The registering of smart home device is shown in Figure 7.

**Figure 8:**
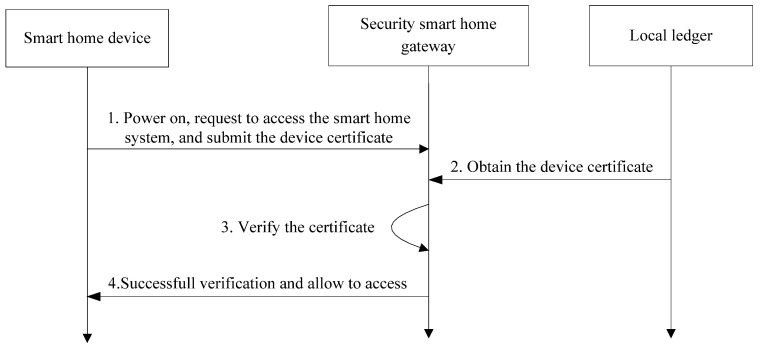
Access control processing of smart home device.

- Step 1: Manufacturers subscribe the device with its certificate for the smart home gateway. If there is no certificate for the device, the manufacturer needs to apply for one from the CA agency (Step 0). For this registering, the certificate and configuration information of smart home devices are necessary.
- Step 2: After receiving the registration request, the security smart home gateway implements certificate authentication by interacting with CA.
- Step 3: After successful authentication, the security smart home gateway writes the certificate and configuration information of the device on the chain. With the detailed process of writing, it needs to go through the endorsement, ordering and committing of other smart home gateways in the network.
- Step 4: After completing the registration, the manufacturer loads the certificate into the device. This certificate is used for accessing the smart home system.

2. Access control:

The access control processing is shown in Figure 8.

- Step 1: After installing and deploying, the smart home device initiates a primary accessing request to the security smart home gateway with its certificate.
- Step 2: When receiving the device’s accessing request, the security smart home gateway obtains the device’s certificate from the local ledger.
- Step 3: The security smart home gateway implements the accessing control by comparing the two certificates. Once the certificate submitted by the smart device is inconsistent with the one stored in the local ledger, the device is not allowed to access the smart home system.
- Step 4: If it succeed, the smart device is allowed to connect.

### 4.3. Security Control

After completing the primary access control, the device connects to the smart home system. During the running processing, the key information in the smart home device is most likely to be attacked by malicious attackers. Therefore, this paper also proposes the security control during the operation of smart home devices, by verifying the integrity of the configuration information. Once the key information of the smart home device is modified, its connection will be interrupted and isolated immediately.

**Figure 9:**
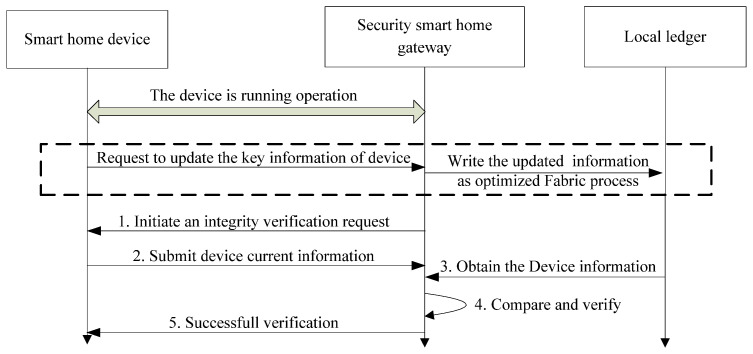
Processing of security control during the operation of the device.

The processing of the security control is shown in Figure 9.

- Step 1: During the operating processing, the security smart home gateway initiates integrity verification irregularly, and requests the current key information of the smart home device.
- Step 2: The smart home device responds to the request and submits the current configuration and port opening information.
- Step 3: After receiving the response, the security smart home gateway downloads the benchmark configuration information from the local ledger, which is written in Fabric when the device is registered.
- Step 4: The security smart home gateway compares the two pieces of information and makes a decision, whether access should continue or not.
- Step 5: For devices where the configuration information has not been modified, they are allowed to access the system continually. If not, the device’s connection will be immediately interrupted and reported to the system administrator and users.

During the operation of the smart home device, the information in the operation system and application software can be updated from time to time. The updated information needs to be written on the chain through the smart home gateway. This section is marked by the dashed box in Figure 8. The specific updating follows the processing of Fabric. Considering a large number of smart devices and frequent updating, the performance optimization for Fabric is of vital importance.

## 5. Experimental Results

The optimized Fabric is deployed in three smart home gateways. One gateway is the orderer, and the others are the endorser and the committer. The client is smart home devices, which is a virtual machine shipped with Fabric SDK.

**Figure 10:**
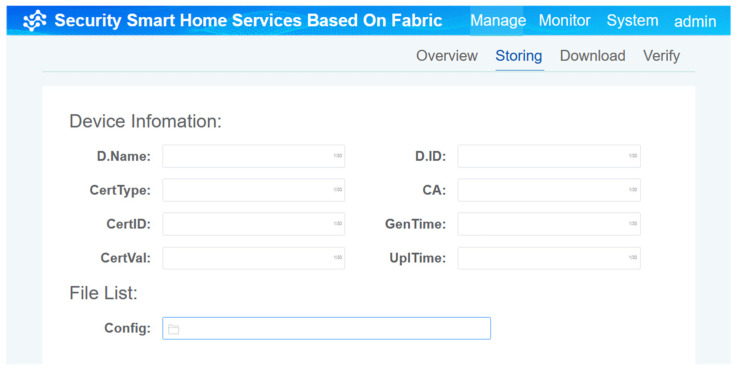
The process of writing device certificates and key information on the chain.

The experimental results are as follows. Figure 10 shows stored device certificates and key information on the chain. This process is initiated during the initial registration of smart home devices and the updating of the running operation. The entries of key information about different devices can be modified. After this operation, the relevant information of the device is stored on the chain.

**Figure 11:**
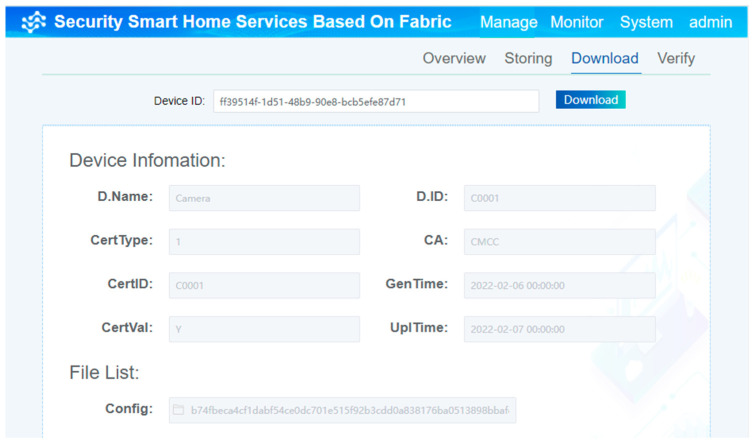
The process of downloading certificates and key information from the chain.

The process of downloading the device certificates and device key information from Fabric by entering a unique device ID is shown in Figure 11. In this process, the information of the device cannot be modified.

**Figure 12:**
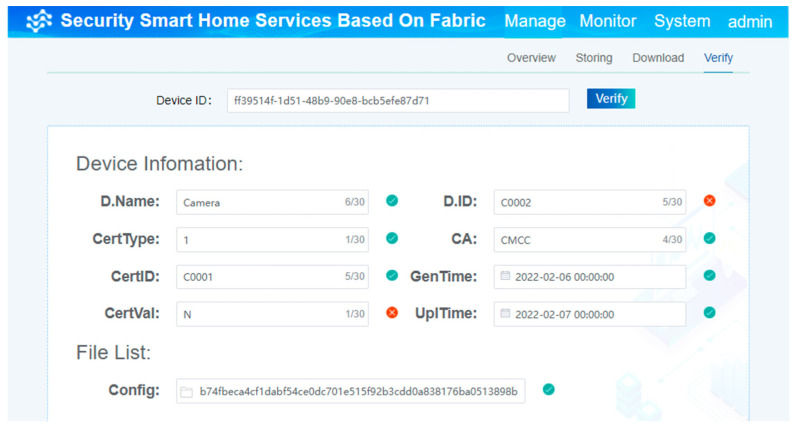
The process of verifying the current information based on the chain.

By comparing the two pieces of information, one is obtained from Fabric and the other is submitted by the device currently, meaning the verification is complete. The specific verification process is shown in Figure 12. Once the two pieces of information are not totally the same as each other, the processing of verification fails. Then, the device is not allowed to access the smart home system.

In the performance experiment, we just focus on the registration process, because the process of access control and security control only involves the download data from Fabric and does not engage in all three stages of Fabric.

**Figure 13:**
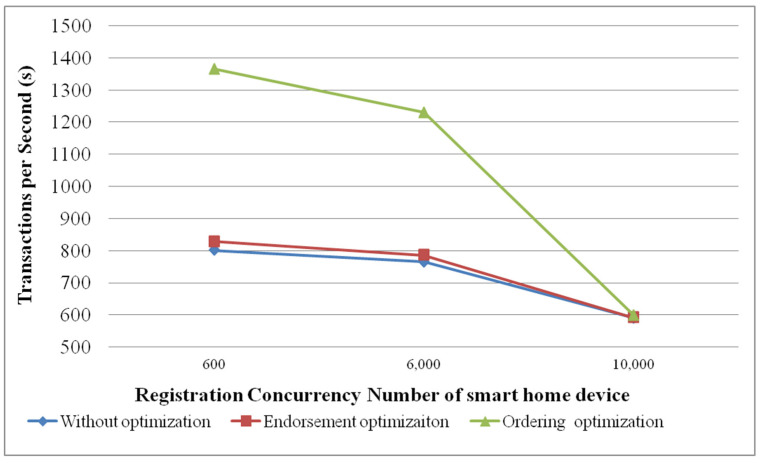
The performance results of smart home device registration based on Fabric.

The performance evaluation is based on the virtual machines, which have 8 cores (Intel(R) Xeon(R) Gold 5118 CPU @ 2.30GHz), 16G memory, 200G hard disk and version 1.4 of Fabric. The smart home device uses jmeter v2.13 software, which is deployed on another virtual machine to simulate a Fabric client and initiate the registration request to Fabric. Comparing the processing performance on the concurrent registration of smart home devices, before and after optimization in Fabric, Figure 13 shows that the performance of Fabric after being optimized has been significantly improved. In Figure 13, as the number of concurrent registrations of smart home devices increases, per second processing performance is decreased. When the number of concurrent registration transactions exceeds 10,000, the optimization effect is not obvious. It is recommended that under such hardware conditions, the optimal number of concurrent registrations for a smart home device should be within 6000.

The performance optimization of the endorsement stage is not obvious in this experiment. This is because the level DB itself has high throughput. However, for non-level DB databases, the effect of concurrency will be more obvious.

The ordering optimization experimental result is reflected in the committing stage. With the optimized ordering mechanism of read-write sets and conflict detection, the workload of the committers is reduced in the committing stage. Figure 13 shows the performance results of the ordering optimization.

In committing stage, the proposed optimization in this paper involves modification of Fabric architecture and the local ledger. As the experimental environment is limited, the experimental result of this proposal has not been analyzed in this paper.

## 6. Conclusions

In this paper, we designed a smart home security control system based on Fabric, by providing efficient access control and security control for smart home devices. With the access control, only the authenticated device can access the smart home service. The security control is active during the operation of smart home devices, by verifying the integrity of the configuration information irregularly. This mechanism prevents smart devices from being attacked during operation and causing security problems for the smart home system. Further, considering the high requirements of performance, we firstly studied the performance optimization for Fabric in this paper. The experimental results show that the smart home security control system works best when the number of concurrent registrations of the smart home device is less than 6000. Regarding the data and application security of smart home services, these need to be further researched in future work.

## Figures and Tables

**Table 1:** The weights of key performance factors in Fabric.

Different Stages	Performance Factors	Weights
Endorsement stage	Complexity of algorithm	high
	Complexity of endorsement	middle
	Performance of database	middle
	The consistency of ledger	middle
	Transaction processing mode	middle
	The performance of peers	low
Ordering stage	Complexity of algorithm	high
	Size of message	high
	Ordering mechanism	high
	The rate of block generation	middle
	Block broadcasting strategy	middle
	The range of broadcasting	low
	The protocol of broadcasting	middle
Committing stage	Complexity of algorithm	high
	The size of block	middle
	Performance of database	high
	The mechanism of verification	high
